# γδ T cells as immunotherapy for malaria: balancing challenges and opportunities

**DOI:** 10.3389/fimmu.2023.1242306

**Published:** 2023-12-06

**Authors:** Ana M. Vigário, Ana Pamplona

**Affiliations:** ^1^ Projecto Medicina, Faculdade de Ciências da Vida, Universidade da Madeira, Funchal, Portugal; ^2^ Instituto de Medicina Molecular João Lobo Antunes, Faculdade de Medicina de Lisboa, Lisboa, Portugal

**Keywords:** gamma-delta (γδ) T lymphocytes, malaria, *Plasmodium*, human malaria, vaccine, immune response, Immunotherapy

## Introduction

Malaria remains a relevant global health problem. In 2021, the WHO estimated 247 million cases worldwide, which led to more than 600,000 deaths, mainly due to severe malaria caused by *Plasmodium falciparum* (1). The different infection outcomes, such as sterile immunity, clinical immunity, and detrimental immune responses observed in severe malaria, are due to the interplay between the parasite and the host. In natural infections, malaria is transmitted after *Plasmodium* sporozoites (Spz) are delivered into the skin during a blood meal of an infected anopheline mosquito and then migrate to the liver. Once inside a hepatocyte, Spz matures and replicates, forming a schizont containing thousands of merozoites, constituting the asymptomatic phase of infection (2). Then, merozoites are released, enter the bloodstream, and infect red blood cells (RBC), initiating the blood-stage infection, which constitutes the symptomatic phase (3).

Therefore, the life cycle of *Plasmodium* involves multiple stages and tissues (skin, liver, and blood), making it difficult to understand the immune responses to the parasite within the vertebrate host. However, creating several opportunities for intervention.

Gamma-delta (γδ) T cells are an immune cell population with increased interest in malaria infection. The singular features of γδ T cells, such as their tissue tropism, pro-inflammatory phenotype, cytotoxic potential, MHC-independent antigen recognition, and properties of natural killer-like cells, make them very interesting targets for therapeutic interventions and vaccine development (*reviewed in* (4)), especially since they have been considered a feasible and promising approach for other clinical situations as cancer immunotherapy (*reviewed in* (5)). Nevertheless, the identification of the antigens recognized by γδ T cells and the subsets involved in protection against malaria, which has been quite challenging, is crucial for understanding the potential clinical application of these cells.

## γδ T cells — of mice and humans

Mouse models have been instrumental in our understanding of malaria parasite biology and the immunopathogenesis of malaria infection and have provided proof of concept for several candidate vaccines and drug discovery. Although mouse models have provided mechanistic insight into γδ T cell biology, human and rodent *Plasmodium* species genomes and mouse and human biology are very different, including on γδ T cells tissue distribution, TCR diversity and effector functions by producing distinct cytokines when stimulated (*reviewed in* (6)). These aspects may difficult the direct translation of knowledge gained from mouse models, such as host–parasite interactions and immune responses, into effective treatment in humans. By other hand, while controlled human malaria infection (CHMI) and human clinical trials provide direct evidence of immune responses to malaria, these studies are usually limited to peripheral blood correlates of protection (*reviewed in* (7)).

## γδ T cells and *Plasmodium* pre-erythrocytic stage – skin an overlooked immune barrier

Although the *Plasmodium* pre-erythrocytic stage has been mainly a designation for the liver stage of infection, we cannot overlook that *Plasmodium* infection begins with the inoculation of Spz in the skin. Several studies performed in mice observed that after inoculation, more than 50% of Spz remain in the skin, particularly in the dermis, and the remaining migrate to the draining lymph nodes (dLN) through lymph vessels or to the liver through blood circulation (8–10). The skin exit of Spz is therefore a bottleneck for successful infection. Indeed, during the skin step, the parasite is more vulnerable to host-immune responses because of the extracellular nature of this stage (11). During this journey, Spz encounters various skin-resident cells, including γδ T cells. Studies in mice and humans have shown that Spz remaining in the dermis are taken up by resident DCs, which can prime CD4+ (12, 13) and CD8+ (14) T-cell responses after migrating to the skin-dLN. These studies showed that skin immune responses impact the induction of T-cell-dependent liver immunity. Interestingly, in a dengue virus infection model, skin mast cell-driven inflammation induced local recruitment and proliferation of γδ T cells (15). In other infections, such as Bacillus Calmette-Guerin (BCG), dermal IL-17-producing γδ T cells increase CD4+ T cell proliferation by inducing neutrophil recruitment, which contributes to antigen delivery to the dLN (16). In addition, in a more recent study using a model of chronic *Trypanosoma brucei* infection, the authors showed that IL-17- producing Vγ6+ cells play a critical role in controlling skin inflammation, likely limiting, either directly or indirectly, dermal IFNγ-mediated CD8+ T cell responses (17). Overall, these studies provide new insights into the role of skin γδ T cell as important players in immunity during infection. Mice lacking canonical dendritic epidermal T cells (DETCs), such as Trgv5-/-Trdv4-/- mice or FVB.Tac mice deficient for Vγ5Vδ1 due to a mutation in Skint1 (18), a butyrophilin-like protein essential for the selective development of the TCR-Vγ5Vδ1-expressing repertoire of murine DETCs (19, 20), or blocking intradermal Skint1 (19), and double Vγ4/6 T cell knockout mice (on an FVB/N genetic background) (17) could be used as strategies to evaluate the relevance of DETCs or dermal γδ T cell populations after Spz inoculation in the skin. Notably, skin γδ T cell can be of particular importance if we want to improve the vaccination strategy for malaria or other infectious diseases using skin-deliverable vaccines (21–24) (*reviewed in* (25)). Thus, a better understanding of the crosstalk between skin γδ T cell subsets and other local cells and the immunological pathways that are triggered after Spz infection is urgently required. The same applies to parasite antigens that can be recognized by skin γδ T cells.

## γδ T cells and *Plasmodium* pre-erythrocytic stage

The human liver is enriched with Vδ2- γδ T subsets, mainly by Vδ1+ but also by Vδ3+, combined with diverse Vγ chains (26). Human Vδ3+ T cells are activated by CD1d-mediated recognition of glycolipids (27) and release IL-17A after activation (27), and Vδ1 TCRs can recognize MICA and MICB ligands expressed by stressed cells (28) and several CD1d- and CD1c-presented glycolipids (26, 29). The intrahepatic γδ T subsets undergo clonal expansion and differentiation in the context of liver virus infection (26). A liver-resident CD27loCD45RAlo subset of Vδ1+ T cells expressing CXCR3 and CXCR6 and producing IFN-γ and TNF-α were identified in humans (26). Interestingly, in a mouse model of HBV infection, liver resident γδ T cells also expressing CXCR3+CXCR6+ produced high levels of IFN-γ, providing protection against acute HBV infection (30). These studies suggest a therapeutic potential of γδ T cells on liver infections (26).

The crosstalk between the liver and the symptomatic blood stages of *Plasmodium* infection has been challenging to study and is thus still poorly understood. A study using a mouse model revealed that IFNγ-producing γδ T cells play a pathogenic role that is strictly dependent on the liver stage of infection, and affecting disease severity (31). This study represents an important step toward understanding the role of γδ T cells and the impact of the liver stage on the pathogenesis of *Plasmodium* infection (31), which was corroborated by a more recent study (32). In this last study, mice were first infected with non-productive Spz that do not transit between the liver and blood stages, followed by low or high doses of infected RBCs two days later (32). In this experimental approach the γδ T cells came into two flavors. On the one hand, protective IL-17-producing γδ T cells expand following low doses of infected RBCs, while IFNγ-producing γδ T cells are induced after high doses of infected RBCs, contributing to disease severity (32). However, it remains to be determined whether a human IL-17-producing γδ T cell counterpart exists. This study highlights the dichotomous nature of γδ T cells in malaria infection. In this context, clarifying the potential dual role of γδ T cells in protection *versus* pathogenesis may provide new avenues for treatment against malaria.

Increased evidence using whole Spz immunizations in humans and mice shows an important role for γδ T cells in protection from subsequent infection (33–36). In a mouse model, induction of γδ T cells after Spz immunization induced protective immunity against parasites in the absence of αβ T cells (35). In another study, a subset of γδ T cells, along with CD8α^+^ DC, was required for the induction of protective CD8+ T cell responses (34). Overall, the mechanism involving γδ T cells-protective role is still unclear, namely whether they act as effector cells or as accessory cells inducing protective CD8+ T cell responses. Importantly, there is still limited knowledge of liver-resident γδ T cells and which subset induces effector CD8+ T cell responses. Understanding the mechanisms of protection by γδ T cells is crucial for developing vaccine strategies against the pre-erythrocytic stage.

## γδ T cells and *Plasmodium* erythrocytic stage

In humans, the accessibility and symptomatic features of the erythrocytic stage make it less challenging to study and implement therapeutic interventions. During this stage, γδ T cells from the peripheral blood and secondary lymphoid organs, particularly the spleen, may encounter parasite antigens or interact with stress-induced molecules expressed by different types of cells in response to infection. Moreover, infected RBCs (iRBCs) are sequestered in the microvasculature of many organs, both in mice and humans (37–39), favoring the interaction of peripheral and tissue-resident γδ T cells with parasite antigens (40).

While Vδ1 cells are the main γδ T cell subset observed in tissues, Vγ9+Vδ2+ are the predominant subset in the peripheral blood of healthy adults (41–44) and may significantly expand during infections (45, 46). Nevertheless, it remains unclear how Vγ9+Vδ2+ T cells are activated and how they contribute to the control of the erythrocytic stage of the parasite. Previous work suggested that soluble molecules, such as phosphoantigens released by mature forms of *P. falciparum*, when cells egress from the iRBCs can activate *in vitro* the Vγ9+Vδ2+ T cells in a contact-independent manner (40). The relevance of this contact-independent activation in controlling circulating iRBC levels *in vivo* is difficult to assess because these molecules are quite diluted in the bloodstream. However, this does not exclude the possibility that this activation mechanism may occur in specific sites, such as the red pulp of the spleen, where iRBCs are retained, or in microvessels where iRBCs can sequester and reduce blood flow (40). Recently, however, a study showed that Vγ9+Vδ2+ T cells recognize, through their γδ TCR, butyrophilin 3A1 (BTN3A1) expressed by all forms of the parasite, except merozoites, in iRBCs (47). This interaction between TCR and BTN3A1 induced the release of both granulysin and granzyme and directly killed iRBCs (47). Vγ9+Vδ2+ T cells also phagocytized and degraded antibody-coated iRBCs in a CD16-dependent manner (47). In fact, peripheral Vγ9+Vδ2+ T cells from *P. falciparum*–infected patients showed an *in vivo* increased surface expression of antigen-presenting cell (APC)-associated markers, upregulated these markers upon stimulation *in vitro* with iRBC, and activated naive CD4+ and CD8+ T cells demonstrating APC capacity (48). A recent report showed that in macaques, a single immunization with a Vγ9+Vδ2+ T cell specific ligand induced a durable memory-like response and amplified IFN-γ responses by other T cell subsets, i.e., CD4+ and CD8+ T cells, reducing *Mycobacterium tuberculosis* pathology and infection (49), an encouraging finding for their potential application in *Plasmodium* infection.

Thus, the role of γδ T cells in the induction and modulation of the adaptive immune response to malaria must be fully understood, as these findings pave the way for their use in therapeutic interventions and vaccine design improvement. In addition to the above features, γδ T cells, when activated, produce different cytokines depending on the subset, ligands, stage of infection, and previous exposure to the antigen. These factors may dictate their functional role, protective or pathogenic, during the erythrocytic stage, making therapeutic intervention more challenging to develop and implement. Notably, cytokines produced by γδ T cells in response to infection may modulate immune memory by influencing the antibody response, which could be very interesting for vaccine designs.

Several studies have reported the expansion of γδ T cells in the blood and spleen of *P. falciparum*-exposed individuals (50–52). In mice, γδ T cells increased in frequency and absolute number in the spleen (52) and lungs after infection, with concomitant increase in absolute numbers of CD4+ and CD8+ T cells, suggesting that γδ T cells could induce the recruitment of T cells after infection (53). A recent mouse study showed that expansion of the M-CSF-producing γδ T cells subset after the acute phase of infection prevented the relapse of parasitemia (54). In CHMI, blood γδ T cells also showed increased responsiveness to *Plasmodium* antigen *in vitro*; this response persisted for more than one year (55). Both studies showed the potential of peripheral blood γδ T cells to prevent chronic *Plasmodium* infections and that *Plasmodium* antigens may induce memory-like γδ T cells. Of note, memory-like γδ T cell responses have been recently described for other infections (56–58). Thus, understanding how these cells are activated may provide clues to new vaccination approaches.

The response of γδ T-cell subsets appears to be multifactorial, depending on the age and ethnicity of the host and malaria endemicity. In studies performed in *P. falciparum* endemic areas, Vδ1+ T cells seem to be the dominant subset (59–61), which do not corroborate the expansion and sustained response of peripheral blood Vγ9+Vδ2+ T cells observed in malaria-unexposed Caucasians (48, 62, 63). Interestingly, the Vδ1+ subset also seems to predominate among healthy individuals in *P. falciparum* endemic areas (59, 61). Recent studies suggest that in malaria-endemic areas, the decreased or unresponsiveness of Vδ2+T cells may be associated with disease tolerance and, therefore, with the development of ‘clinical immunity’ induced by successive malaria episodes (64, 65). Clarifying these mechanisms is essential to take advantage of γδ T cells for therapeutic or preventive interventions.

In a study evaluating whole-organism *P. falciparum* Spz vaccine in Malian adults, the Vδ2+T cell subset expanded after vaccination and not Vδ1+ T cell subset (34). Understanding why we observed differences in γδ T cell subset responders between endemic and non-endemic malaria populations and between children and adults from endemic regions requires further evaluation. Interestingly, RTS,S phase 3 trials in African children did not detect changes in total γδ T cell frequencies after vaccination and detected negligible cytokine production by these cells upon *in vitro* circumsporozoite protein (CSP) stimulation (66). Future studies should characterize more specifically γδ T cell subsets, such as Vδ2+ or Vδ1+ T cells, and not total γδ T-cells, and evaluate whether these subsets correlate with protection.

## Concluding remarks

**Figure 1:**
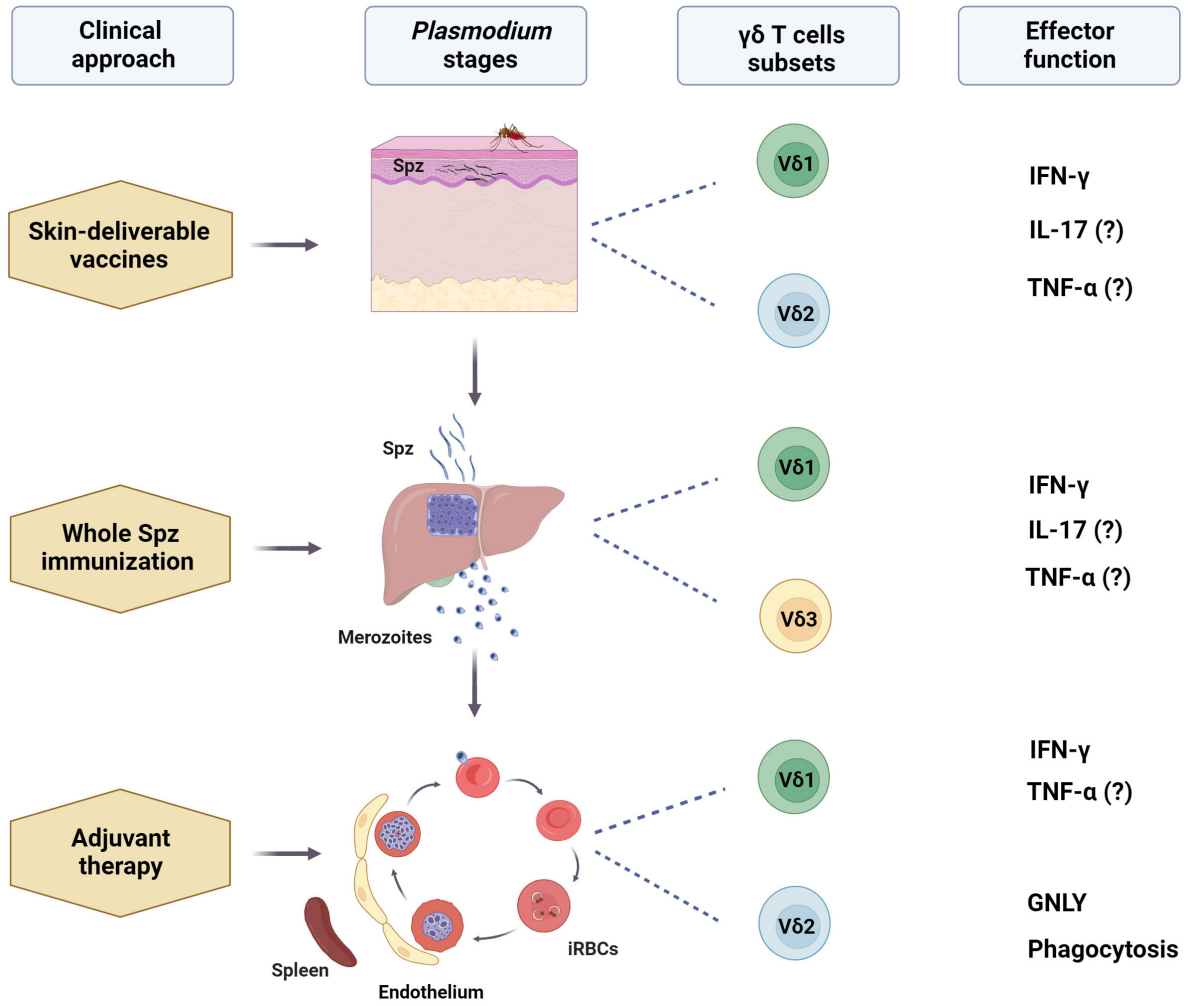
Potential use of human γδ T cells-based immunotherapy for malaria. After a mosquito bite, γδ T cells, especially Vδ1+, can engage with sporozoites (Spz) in the dermis, as well as recruited circulating Vδ2+ T cells. These cells can interact with other local immune cells and be activated by mosquito saliva components, malarial antigens, and stress-induced molecules. Skin-deliverable vaccines may enhance these interactions, promoting the subsequent development of adaptive immune responses, which, in turn, may increase Spz vaccination efficacy. Additionally, including γδ T cell-activating molecules in skin-deliverable anti-malaria vaccines may increase their effectiveness. After leaving the skin, sporozoites quickly reach the liver, where Vδ1+ and Vδ3+ are the primary subsets of γδ T cells. Studies on Spz immunization have suggested that liver-resident γδ T cells contribute to the development of protective immunity against the parasite. This finding emphasizes the promise of these cells in increasing the effectiveness of malaria vaccines. Furthermore, circulating Vδ2+ T cells may migrate to the liver post-immunization. During the blood stage, the most mature forms of the parasite may interact with circulating and splenic Vδ2+ and Vδ1+ T cells and may be implicated in the control of parasitemia. Activating γδ T cells with specific ligands may improve the anti-parasitic effects of conventional therapies, acting as an adjuvant therapy, and ultimately induce a memory-like γδ T cell response. However, the specific γδ T cell subset, ligands, and effector functions responsible for the potential protective role of γδ T cells in malaria remain unclear. (?) means "unclear evidence in human malaria". Created with BioRender.com.

In summary, the use of strategies involving γδ T cells in the context of malaria is a promising but challenging field of research because of the complexities of malaria infection. Moreover, most immunotherapy interventions are costly, especially those designed for cancer, and malaria is a widespread infectious disease in low- or middle-income countries. Nevertheless, several approaches should be pursued for the use of γδ T cells in malaria (**Figure 1**).

One approach may involve the use of ligands for the activation of γδ T cells to induce an antiparasitic effect in infected individuals (40, 47), which may be combined with conventional anti-malaria therapies. Identifying specific ligands that can selectively activate γδ T subsets associated with parasite elimination or protection against severe manifestations of the disease and that have already been used in the contexts of other infections (67, 68) and cancer (69–71) is a challenging task. γδ T cells can also be targeted in vaccine strategies. This can be achieved by incorporating specific γδ T cell ligands into vaccines to induce a stronger adaptive immune response. γδ T cells can act as antigen-presenting cells, contributing to the activation of the adaptive immune response and thus increasing the host’s effectiveness against malaria (48). The activation of γδ T cells can also be potentiated using specific adjuvants that stimulate innate immune cells, which in turn can promote the recruitment, proliferation, or memory-like phenotype on γδ T cells (15, 56–58). This can be combined with vaccine strategies to enhance the effectiveness of the immune response to the malaria parasite.

The fact that γδ T cells populate several tissues and thus may interact with the various forms of Plasmodium parasites during each specific stage of the parasite life cycle dramatically increases the possibility of intervention using these cells. However, progress depends on the full understanding of the interactions between the parasite, host cells, γδ T cells, and other immune cells, as well as the clarification of which γδ T cell subsets correlate with immune protection and which ligands are most effective in activating these subsets.

## Author contributions

AMV and AP conceived and wrote the manuscript. All authors contributed to the article and approved the submitted version.
