# Erratum: γδ T cells as immunotherapy for malaria: balancing challenges and opportunities

**DOI:** 10.3389/fimmu.2024.1364914

**Published:** 2024-01-11

**Authors:** Frontiers Production Office

**Affiliations:** Frontiers Media SA, Lausanne, ;Switzerland

**Keywords:** gamma-delta (γδ) T lymphocytes, malaria, Plasmodium, human malaria, vaccine, immune response, Immunotherapy

Due to a production error, several words and symbols were omitted from the published article.

A correction has been made to the section “γδ T cells and *Plasmodium* erythrocytic stage”:

In the sentence, “Previous work suggested that soluble molecules, such as phosphoantigens released by mature forms of P. falciparum, when parasites egress from the iRBCs, can activate *in vitro* the Vγ9+Vδ2+ T in a contact-independent manner,”. The word “cells” is missing after Vγ9+Vδ2+T. It should be Vγ9+Vδ2+ T cells.

After the second reference 47 in the sentence, “Vγ9+Vδ2+ T cells also phagocytized and degraded antibody-coated iRBCs in a CD16-dependent manner,”. The letter V is missing after the Vγ9+. It should be Vγ9+Vδ2+ T cells.

The publisher apologizes for these mistakes. The original version of this article has been updated.

